# Extraction of gall bladder via umbilical port versus subxiphoid port for laparoscopic cholecystectomy in Pakistan: A systematic review and meta-analysis

**DOI:** 10.1097/MD.0000000000045963

**Published:** 2025-11-21

**Authors:** Syeda Zuha Sami, Sania Nisar, Alisha Ahmed, Inibehe Ime Okon

**Affiliations:** a Department of Internal Medicine, Shaheed Mohtarma Benazir Bhutto Medical College Lyari, Karachi, Pakistan; b Department of Internal Medicine, Aga Khan University, Karachi, Pakistan; c Department of Internal Medicine, Jinnah Sindh Medical University, Karachi, Pakistan; d Department of Research, Medical Research Circle (MedReC), Bukavu, DR Congo.

**Keywords:** epigastric port, gallbladder extraction, laparoscopic cholecystectomy, meta-analysis, port-site infection, postoperative pain, subxiphoid port, umbilical port

## Abstract

**Background:**

Gallbladder retrieval site in laparoscopic cholecystectomy (LC) may influence postoperative outcomes, yet there is limited consensus on the optimal port for extraction. This systematic review and meta-analysis aimed to compare the umbilical port (UP) versus epigastric/subxiphoid port (EP/SP) in terms of clinical efficacy and safety.

**Methods:**

This review was conducted according to Preferred Reporting Items for Systematic Reviews and Meta-Analyses guidelines. A comprehensive search of PubMed, Google Scholar, and the Cochrane Library was performed through June 2025. Only randomized controlled trials from Pakistan comparing EP and UP for LC were included. Outcomes assessed included postoperative pain at multiple intervals, retrieval time, port-site infection, and hospital stay. Data were synthesized using Review Manager (RevMan) 5.4.1 with random-effects models, and heterogeneity was assessed via *I*² statistics. Risk of bias was evaluated using Cochrane risk of bias (RoB 2.0).

**Results:**

Nine randomized controlled trials involving 1338 patients (672 EP, 666 UP) were analyzed. UP retrieval was associated with significantly lower postoperative pain at 1, 6, 24, and 48 hours. Retrieval time was shorter in the UP group (mean difference: –1.34 minutes; 95% confidence interval: –2.40 to –0.28; *P* = .01). Hospital stay was also reduced with UP (mean difference: –0.73 days; 95% confidence interval: –1.31 to –0.16; *P* = .01). Port-site infection rates showed no significant difference (risk ratio = 1.23; *P* = .90). Sensitivity analyses supported the robustness of key findings.

**Conclusion:**

UP retrieval in LC offers favorable early postoperative outcomes, including reduced pain and shorter hospital stay, compared to the subxiphoid port. These results support UP as a potentially preferred approach.

## 1. Introduction

Laparoscopic cholecystectomy (LC) has become an optimal standard of treatment for gallbladder diseases, revolutionizing the field of minimally invasive surgeries.^[1,2]^ LC is associated with decreased discomfort, pain, and infection at the surgical site; a lower risk of developing an incisional hernia; fewer cases of pneumonia or cardiopulmonary complications; a shorter hospital stay; and improved quality of life, mortality, and morbidity.^[3,4]^ The routine indications of LC include symptomatic cholelithiasis, acalculous cholecystitis, biliary dyskinesia, gallstone pancreatitis, and other gallstone polyps and masses. Conditions such as metastasis, poor tolerance to general anesthesia, and refractory coagulopathy are all contraindications for LC treatment.^[5]^

A typical LC is performed using 3 or 4 ports of entry into the abdomen. Multiport LC has proven to be safe and effective, but reduced-port or 1-port LC is actively being applied as it is effective for better cosmetic outcomes. Single-port LC increases the complexity of surgery and complications such as risk of bile duct spillage or injury, incisional hernia, and increased cost.^[6,7]^ Following the identification, the cystic duct and artery are dissected, divided, and ligated using clips. Subsequently, the gallbladder is separated from the liver bed and removed. The extraction of the gallbladder through one of the ports is a critical step in the LC procedure, as gallbladder perforation remains the leading cause of spillage of stones and bile during surgery, increasing the risk of wound site infection and morbidity.^[8,9]^ Port-site morbidity is reported as one of the challenging complications compromising the efficacy of LC.^[4,10]^ Port-site complications and morbidity, such as bleeding, infection, and hernia, are being reduced by using various extraction techniques, including retrieval devices such as endo bags or introducing a close approach of pneumoperitoneum.^[8,11]^

The gall bladder can either be extracted through the epigastric port (EP) or the umbilical port (UP).^[12,13]^ The port of retrieval is generally selected based on the surgeon’s preference. Some surgeons tend to avoid UP extraction as it is the most common location of port-site hernia.^[14,15]^ The choice of optimal port-site for gallbladder extraction in LC is still being debated in the literature. A pooled analysis found that epigastric and UP retrieval resulted in comparable postoperative pain outcomes.^[16]^ While another reported fewer postoperative pain incidence in UP retrieval.^[17]^ Although the UP reports a higher risk of developing a port-site hernia, the procedure time could be longer.^[16]^

Due to diverse surgical practices, limited available national guidelines and resources, retrieval technique inconsistencies are more frequent in low- to middle-income countries such as Pakistan. A pooled analysis is required for assessment of data available across Pakistan to determine an optimal port-site in terms of clinical outcomes such as postoperative pain, port-site hernia, and surgical site infection. Hence, we conducted a systematic review and meta-analysis of all randomized controlled trials (RCTs) conducted in Pakistan to compare the impact and reliability of gallbladder retrieval through the UP versus EP.

## 2. Materials and methods

This systematic review and meta-analysis is conducted according to the Preferred Reporting Items for Systematic Review and Meta-Analysis (PRISMA) guidelines^[18]^ with its protocol registered in International Prospective Register of Systematic Reviews (CRD420251164890).

### 2.1. Data source and search strategy

A comprehensive literature search was conducted on PubMed Central, Google Scholar, and The Cochrane Library from inception to June 2025. Bibliographies of relevant articles were also searched to make sure no studies were missed. No filters were applied on language, sample size, year of publication, author name, and institution of publication. The electronic search strategy was conducted using the following keywords: “Laparoscopic cholecystectomy” AND “Umbilical Port” OR “Transumbilical Port” AND “Subxiphoid Port” OR “Epigastric Port.” A detailed description of the complete search strategy applied for each database is given in Table S1, Supplemental Digital Content, https://links.lww.com/MD/Q663.

### 2.2. Study selection and eligibility criteria

All articles retrieved from the systematic search were exported to EndNote X9 Reference Manager (Clarivate Analytics) where duplicates were removed among different online databases. Two independent reviewers performed an initial screening of the remaining articles based on the title and abstract that met the study population. Finally, full texts were evaluated for relevance. Any discrepancies were resolved by discussion with a third reviewer. The search was restricted to the following inclusion criteria: studies that compared EP versus UP for LC, patients above 18 years, RCTs conducted, and outcomes of interest were reported which included: postoperative pain, mean time taken for retrieval, port-site infection, port-site hernia, operative time, extending of port incision, unsatisfactory port-site scaring, and postoperative hospital stay. The exclusion criteria were: conference papers, abstracts, case series, case reports duplicated studies, single-arm studies, age below 18 years, studies that lacked a comparator group, and studies that did not report any outcome of interest.

### 2.3. Data extraction

Two reviewers independently extracted the data from selected studies. The general characteristics of the included studies were extracted and entered on the Microsoft Excel sheet the authors created. The following data was extracted from each study: study name and year, country, sample size, the number of patients in each group (EP vs UP), follow-up period, general patient characteristics (age, male percentage, female percentage, body mass index, diabetes mellitus, hypertension, smoking, alkaline phosphatase, pethidine requirement, ketorolac requirement, American Society of Anesthesiologists scores, gall stones and gall bladder polyps), and all outcomes of interest. Continuous outcomes reported as median with interquartile ranges were converted to mean and standard deviation using Wan’s method.

### 2.4. Quality assessment and risk of bias assessment

**Figure 1.**
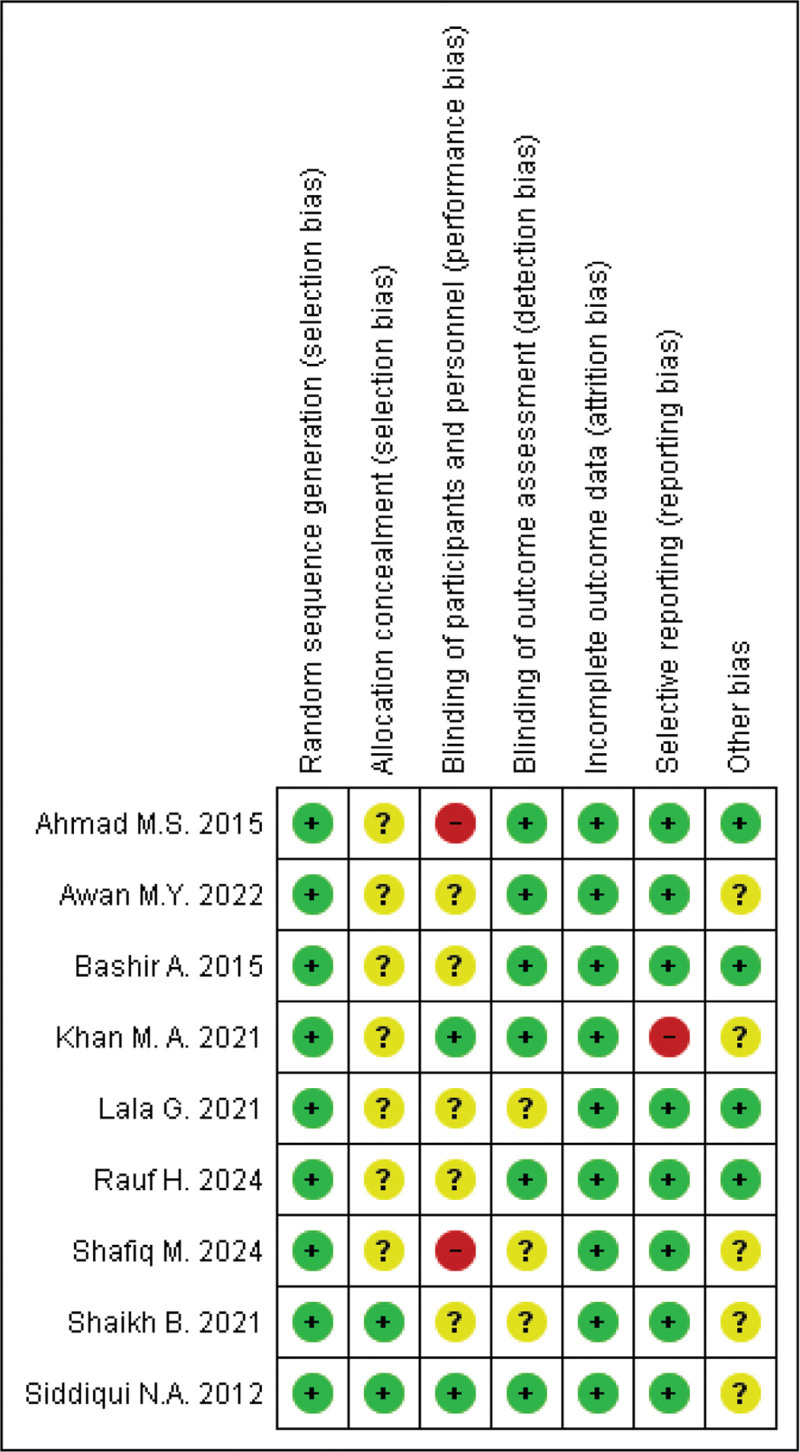
Risk of bias summary.

Two independent reviewers performed a quality assessment of the RCTs by Cochrane Risk of Bias (RoB 2.0) tool. The following sources of bias were considered: selection bias, performance bias, attrition bias, detection bias, reporting bias, and other potential sources of bias. The overall risk of bias for each study was evaluated and rated: low, unclear, or high. Any disagreement was resolved by a third reviewer. The risk of bias summary and graphical assessment is detailed in Figures 1 and S1, Supplemental Digital Content, https://links.lww.com/MD/Q663 respectively.

### 2.5. Statistical analysis

For statistical analysis, Review Manager (RevMan, Version 5.4.2; The Cochrane Collaboration, London, UK) was used. Dichotomous data was used to drive the risk ratio and corresponding 95% confidence intervals (95% CIs) and similarly, for the continuous outcome, mean difference (MD) was obtained and their 95% CIs by using the random-effects model. The probability value of *P* < .05 was considered statistically significant. Higgins *I*² was used to measure heterogeneity.^[19,20]^ The value of *I*² = 25% to 50% was regarded as mild heterogeneity, 50% to 75% as moderate heterogeneity, and >75% as high heterogeneity. When we noticed high heterogeneity, we performed a leave-one-out analysis to rule out the cause. For better understanding of the findings, subgroup analysis was also performed. Funnel plots were constructed for the outcomes which included >10 studies to check for any publication bias. Comprehensive meta-analysis (version 3.3.070) was used to perform meta-regression to explore sources of heterogeneity and funnel plots were used to assess publication bias. These methodological approaches ensured that our results were accurate and could be interpreted with confidence and help minimize the impact of confounding factors.

## 3. Results

### 3.1. Study selection

In our meta-analysis of 9 RCTs,^[21–29]^ we adhered to PRISMA guidelines. Initially, we identified 1318 records from PubMed (252), Google Scholar (915), and Cochrane Library (151). After removing 312 duplicate records, 1006 records underwent screening based on abstracts and titles, resulting in the exclusion of 520 records. We sought to retrieve 486 reports for full-text assessment, out of which 17 were not retrieved and 460 were excluded due to reasons such as not providing relevant data (62), being review articles (13), non-comparative studies (80), or inconsistent reporting of outcomes (305). Ultimately, 9 RCTs met the inclusion criteria and were included in our meta-analysis. The PRISMA flowchart summarizes our screening process (Fig. S2, Supplemental Digital Content, https://links.lww.com/MD/Q663).

### 3.2. Study characteristics

A total of 9 RCTs^[21–29]^ comprising 1338 patients (672 in EP vs 666 in UP). The mean age of patients in EP is 43.9 years, and 43.5 years in UP. Several baseline characteristics were extracted and assessed. A summary of baseline characteristics is given in Table 1.

**Table 1:** Characteristics of included studies.

Characteristics of included studies																
Author, year	Study place	Follow-up	Patient population (n)		Avg. age (yr), mean (SD)		Male: female		BMI (kg/m^2^), mean (SD)		ASA score I		ASA score II		Duration of surgery (min)	
			EP	UP	EP	UP	EP	UP	EP	UP	EP	UP	EP	UP	EP	UP
Ahmad M.S. 2015	Lahore, Pakistan	24 h	30	30	48.5 ± 7.4	48 ± 5.7	9:21	15:15	–	–	–	–	–	–	–	–
Awan M.Y. 2022	Abbottabad, Pakistan	–	53	53	47.19 ± 13.44	44.6 ± 15.5	17:36	22:31	–	–	–	–	–	–	–	–
Bashir A. 2015	Lahore, Pakistan	24 h	50	44	47.94 ± 7.394	46.84 ± 5.640	17:33	25:19	–	–	–	–	–	–	–	–
Khan M.A. 2021	Gambat, Pakistan	1 mo	30	30	47.94 ± 7.394	46.84 ± 5.640	11:19	13:17	–	–	–	–	–	–	–	–
Lala G. 2021	Lahore, Pakistan	48 h	35	35	33.86 + 6.01	36.17 + 5.88	14:21	13:22	–	–	–	–	–	–	–	–
Rauf H. 2024	Lahore, Pakistan	72 h	50	50	43.40 ± 15.21	44.96 ± 16.63	17:33	29:21	29.96 ± 4.74	31.05 ± 5.03	28	24	22	26	–	–
Shafiq M. 2024	Karachi, Pakistan	48 h	50	50	45 ± 15.2	44.74 ± 16.1	25:25	26:24	30.6 ± 5.8	28.8 ± 6.8	20	9	16	24	75.26 ± 26.74	77.56 ± 26.7
Shaikh B. 2021	Sukkur, Pakistan	5 d	314	314	38.560 ± 6.23	38.875 ± 8.11	91:223	119:195	27.437 ± 5.04	29.973 ± 5.12	–	–	–	–	48.920 ± 8.67	50.656 ± 8.41
Siddiqui N.A. 2012	Karachi, Pakistan	36 h	60	60	42.5 ± 10.7	40.6 ± 12.6	13:47	15:45	–	–	–	–	–	–	52.5 ± 12.1	56.7 ± 13.8

Avg = average, BMI = body mass index, EP = epigastric port, SD = standard deviation, UP = umbilical port.

### 3.3. Risk of bias of included studies

The risk of bias is assessed using the Cochrane Risk of Bias (RoB 2.0) tool for RCTs (Fig. 1).

## 4. Primary outcomes

### 4.1. Postoperative pain at 24 hours

**Figure 2.**
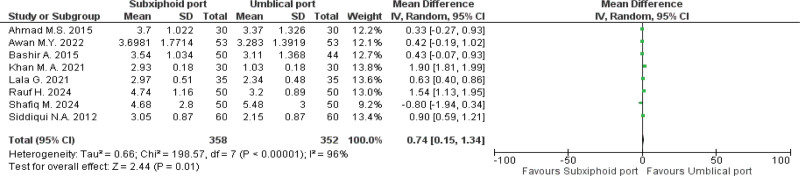
Forest plot of postoperative pain at 24 h. CI = confidence interval, SD = standard deviation.

A total of 710 patients (358 in the subxiphoid port group and 352 in the UP group) were analyzed from 8 studies reporting this outcome. The pooled analysis revealed that postoperative pain scores were significantly higher in the subxiphoid port group compared to the UP group, with an MD of 0.74 [0.15, 1.34]; *P* = .01 (Fig. 2). However, high heterogeneity (*I*^2^ = 96%) is apparent, for which a sensitivity analysis was performed using the leave-one-out approach; yet, no significant change in heterogeneity was observed.

### 4.2. Mean time taken for retrieval

**Figure 3.**
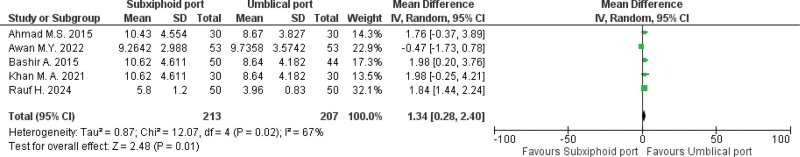
Forest plot of mean time taken for retrieval. CI = confidence interval, SD = standard deviation.

Five studies with a total of 420 patients (213 in the subxiphoid port group and 207 in the UP group) were included in the analysis of the mean time taken for retrieval. The pooled results showed that the subxiphoid port approach was associated with a significantly longer retrieval time compared to the UP, with an MD = 1.34 (95% CI: 0.28, 2.40); *P* = .01 (Fig. 3). Moderate-to-high heterogeneity was observed (*I*² = 67%), for which sensitivity analysis was performed using the leave-one-out approach, as a result of which a change in heterogeneity was noticed.

### 4.3. Leave-one-out of the analysis

**Figure 4.**
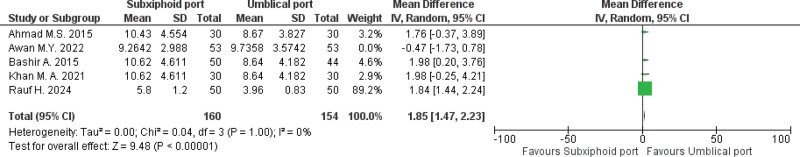
Forest plot of leave-one out analysis of mean procedural time. CI = confidence interval, SD = standard deviation.

Since high heterogeneity was identified, a leave-one-out analysis was performed by ruling out Awan et al^[22]^ which dropped the heterogeneity to 0. The figure of the forest plot of leave-one-out analysis with MD: 1.85 [95% CI: (1.47, 2.23); *P* < .00001, *I*² = 0%] is displayed in Figure 4.

## 5. Secondary outcomes

### 5.1. Postoperative pain at 48 hours

A total of 170 patients were analyzed from 2 studies that reported this outcome. We found that postoperative pain scores at 48 hours were significantly higher in the subxiphoid port group compared to the UP group (MD = 0.54 [0.31, 0.77], *P* < .00001). Pooled data were homogeneous (*P* = .67); *I*² = 0%, indicating no statistical heterogeneity across the included studies (Fig. S3, Supplemental Digital Content, https://links.lww.com/MD/Q663).

### 5.2. Postoperative pain at 12 hours

A total of 450 patients were analyzed from 5 studies that reported postoperative pain at 12 hours. No statistically significant difference in pain scores between the subxiphoid and UP groups (MD = 0.32 [−0.70, 1.35]; *P* = .54; Fig. S4, Supplemental Digital Content, https://links.lww.com/MD/Q663). Pooled data were highly heterogeneous (*P* < .00001); *I*² = 99%, for which sensitivity analysis was performed using the leave-one-out approach, and yet there was no significant change observed in heterogeneity.

### 5.3. Postoperative pain at 6 hours

A total of 225 patients from 5 studies were analyzed for postoperative pain at 6 hours. The pooled analysis demonstrated that pain scores were significantly higher in the subxiphoid port group compared to the UP group, with an MD = 1.08 (95% CI: 0.78, 1.37); *P* < .00001 (Fig. S5, Supplemental Digital Content, https://links.lww.com/MD/Q663). However, substantial heterogeneity was observed (*I*² = 77%), for which sensitivity analysis was performed using the leave-one-out approach, as a result of which a change in heterogeneity was noticed.

### 5.4. Leave-one-out of the analysis

Since high heterogeneity was identified, a leave-one-out analysis was performed by ruling out Lala et al,^[25]^ which dropped the heterogeneity from high to mild. Figure of forest plot of leave-one-out analysis with MD: 1.30 [95% CI (1.22, 1.38); *P* < .00001, *I*² = 03%] is displayed in supporting information (Fig. S6, Supplemental Digital Content, https://links.lww.com/MD/Q663).

### 5.5. Postoperative pain at 1 hour

A total of 390 patients from 4 studies were included in the analysis of postoperative pain scores at 1 hour. The pooled results revealed that pain scores were significantly higher in the subxiphoid port group compared to the UP group, with a MD = 0.98 (95% CI: 0.35, 1.61); *P* = .002 (Fig. S7, Supplemental Digital Content, https://links.lww.com/MD/Q663). However, heterogeneity was high (*I*² = 84%), for which sensitivity analysis was performed using the leave-one-out approach, as a result of which a change in heterogeneity was noticed.

### 5.6. Leave-one-out of the analysis

Since high heterogeneity was identified, a leave-one-out analysis was performed by ruling out Shafiq et al,^[27]^ which dropped the heterogeneity from high to moderate. Figure of forest plot of leave-one-out analysis with MD: 1.30 [95% CI: (0.87, 1.74); *P* < .00001, *I*² = 70%] is displayed in Figure S8, Supplemental Digital Content, https://links.lww.com/MD/Q663.

### 5.7. Port-site infection

A total of 698 patients were analyzed from 2 studies reporting on port-site infection. The pooled analysis showed no significant difference in the risk of port-site infection between the subxiphoid and UP groups (risk ratio = 1.23; 95% CI: 0.05–30.17; *P* = .90; Fig. S9, Supplemental Digital Content, https://links.lww.com/MD/Q663). Additionally, the heterogeneity test performed regarding the variation in study outcomes between studies showed (χ^2^ = 8.04, df = 1, *P* = .005), indicating high heterogeneity (*I*^2^ = 88%), but sensitivity analysis cannot be performed due to insufficient studies showing port-site infection.

### 5.8. Postoperative hospital stay

A total of 170 patients were included from 2 studies reporting on postoperative hospital stay. The pooled analysis demonstrated that hospital stay was significantly longer in the subxiphoid port group compared to the UP group (MD = 0.73 days; 95% CI: 0.16–1.31; *P* = .01; Fig. S10, Supplemental Digital Content, https://links.lww.com/MD/Q663). Moderate heterogeneity was observed (*I*² = 47%), but we could not perform sensitivity analysis to assess the risk of bias by the leave-one-out method, because there are not sufficient studies indicating postoperative hospital stay, which could induce some bias in results.

## 6. Discussion

From the RCTs conducted in Pakistan, this meta-analysis shows that the retrieval of the UP may be more advantageous compared to the subxiphoid retrieval for LC, particularly in the early postoperative period. Patients who underwent retrieval through the UP reported lower pain scores across multiple time points, indicating a consistent trend toward improved postoperative comfort. These findings are in agreement with those reported by Kulkarni.^[16]^

Additionally, the anatomical characteristics of the umbilical region may contribute to these outcomes providing a possible explanation for the observed benefits. The umbilicus contains fewer nerve endings and less dense musculature compared to the subxiphoid area, which may result in reduced tissue trauma and nociceptor stimulation during specimen extraction. Hajibandeh similarly reported improved pain outcomes with umbilical extraction, without a corresponding increase in adverse events.^[17]^

Although port-site infection rates were comparable between the 2 techniques in our dataset, existing literature indicates mixed findings. For Example, Kaya reported a decreased incidence of port-site complications, including infection, in patients who underwent umbilical retrieval.^[9]^ However, due to the low number of infection events and limited follow-up duration in many of the included trials, definitive conclusions regarding infection risk remain elusive. Furthermore, Li observed that patients with umbilical retrieval had higher risks of incisional hernia, especially those with predisposing factors, highlighting the importance of meticulous fascial closure and individualized patient selection.^[15]^

In addition to the infection risk considerations, operative efficiency also appeared to favor the umbilical approach. Furthermore, according to most of the included studies it was found that retrieval through the UP was associated with reduced extraction time and shorter hospital stays. These findings align with Lee et al findings, who described smoother intraoperative handling and improved recovery timelines with umbilical access.^[30]^ This suggests potential benefits for patients as well as for surgical workflow and healthcare resource utilization.

## 7. Limitations

Nonetheless, there are notable limitations to consider, such as the lack of long-term follow-up data in the included studies, making it difficult to assess outcomes such as port-site hernia formation, patient satisfaction with cosmetic results, or recurrence of complications. As suggested in previous observational studies, the technique of fascial closure at the umbilical site plays a crucial role in minimizing the risk of postoperative herniation. All in all, standardized outcome measures, longer follow-up durations, and consistent reporting on postoperative complications such as hernia, infection, and cosmetic satisfaction should be included by further studies to provide clearer clinical guidance and strengthen the evidence base.

## 8. Conclusion

In conclusion, when compared to subxiphoid-port retrieval in LC, umbilical-port retrieval appears to provide favorable early postoperative outcomes. Particularly, in terms of reduced pain and shorter hospital stay. Both, anatomical considerations and current clinical evidence, indicate the UP as a potentially preferred choice in standard surgical practice. However, the generalizability of these findings is limited by the lack of robust long-term data, specifically with relation to port-site hernia, cosmetic satisfaction, and recurrence. Until there is a considerable amount of evidence available, careful patient selection, meticulous surgical technique, and ongoing evaluation of outcomes remain essential to ensure optimal results. It is recommended that large-scale, multicenter trials be conducted in the future to confirm these findings and back evidence-based surgical recommendations.

## Author contributions

**Conceptualization:** Syeda Zuha Sami, Sania Nisar.

**Data curation:** Syeda Zuha Sami, Sania Nisar, Alisha Ahmed, Inibehe Ime Okon.

**Formal analysis:** Sania Nisar.

**Funding acquisition:** Inibehe Ime Okon.

**Investigation:** Inibehe Ime Okon.

**Methodology:** Syeda Zuha Sami, Sania Nisar.

**Project administration:** Syeda Zuha Sami.

**Supervision:** Inibehe Ime Okon.

**Validation:** Syeda Zuha Sami, Sania Nisar.

**Visualization:** Alisha Ahmed.

**Writing – original draft:** Syeda Zuha Sami, Sania Nisar, Alisha Ahmed.

**Writing – review & editing:** Inibehe Ime Okon.

## Supplementary Material


