# Supplementary material for: Extraction of gall bladder via umbilical port versus subxiphoid port for laparoscopic cholecystectomy in Pakistan: A systematic review and meta-analysis
Source: Medicine (Baltimore). 2025 Nov 21;104(47):e45963. doi: 10.1097/MD.0000000000045963 (PMC12643762; doi:10.1097/MD.0000000000045963)
Supplement: Supplementary file 1 [file medi-104-e45963-s001.pdf]

Database	Database Search string	Number of articles retrieved
PubMed	(((("cholecystectomy, laparoscopic"[MeSH Terms] OR ("cholecystectomy"[All Fields] AND "laparoscopic"[All Fields]) OR "laparoscopic cholecystectomy"[All Fields] OR ("laparoscopic"[All Fields] AND "cholecystectomy"[All Fields])) AND (("umbilicus"[MeSH Terms] OR "umbilicus"[All Fields] OR "umbilical"[All Fields]) AND "Port"[All Fields])) OR (("transumbilical"[All Fields] OR "transumbilically"[All Fields]) AND "Port"[All Fields])) AND (("subxiphoid"[All Fields] OR "subxiphoidal"[All Fields]) AND "Port"[All Fields])) OR (("epigastric"[All Fields] OR "epigastrical"[All Fields]) AND "Port"[All Fields])	252
Google Scholar	(Laparoscopic cholecystectomy) AND (Umbilical Port OR Transumbilical Port) AND (Subxiphoid Port OR Epigastric Port)	915
Cochrane Library	(Laparoscopic cholecystectomy) AND (Umbilical Port) OR (Transumbilical Port) AND (Subxiphoid Port) OR (Epigastric Port)	151

Supplementary Table 1: Search strategy table

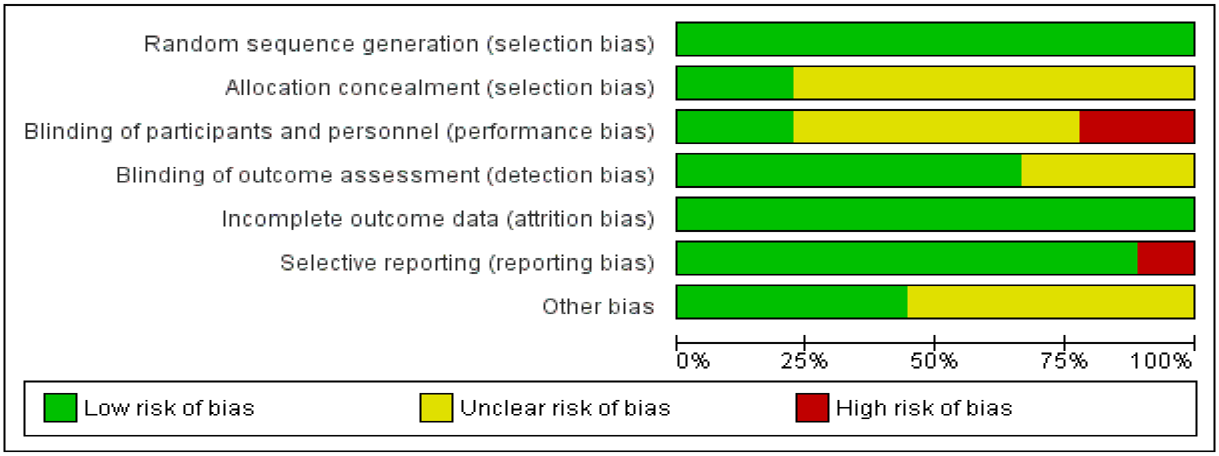

Figure S1: Risk of bias graph

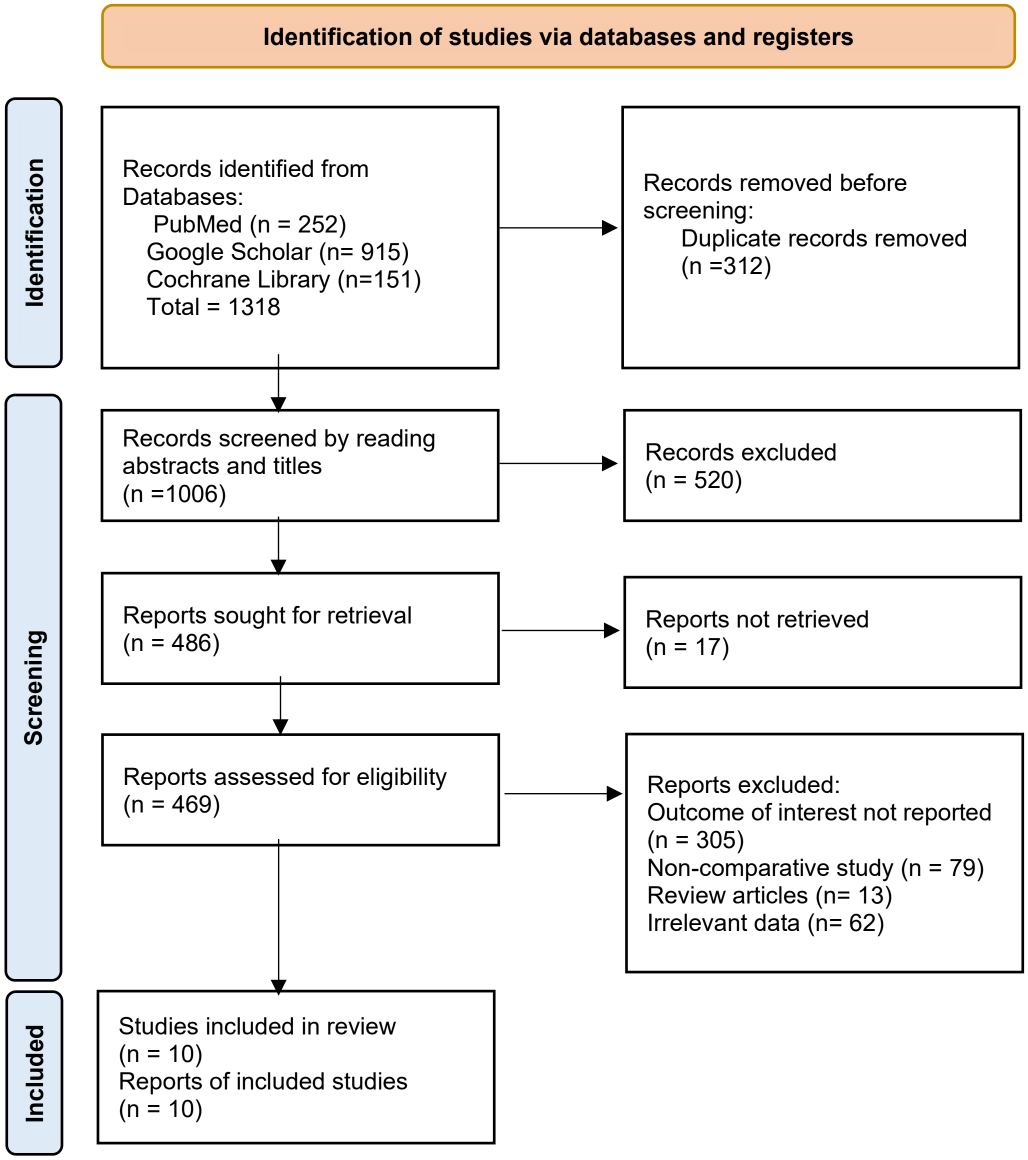

Figure S2. PRISMA flowchart

	Subxiphoid port			Umblical port				Mean Difference
Study or Subgroup	Mean	SD	Total	Mean	SD	Total	Weight	IV, Random, 95% CI
Lala G. 2021	2.49	0.56	35	1.94	0.42	35	96.6%	0.55 [0.32, 0.78]
Shafiq M. 2024	4.82	3.4	50	4.54	2.9	50	3.4%	0.28 [-0.96, 1.52]
**Total (95% CI)**			**85**			**85**	**100.0%**	**0.54 [0.31, 0.77]**

Heterogeneity: Tau² = 0.00; Chi² = 0.18, df = 1 (P = 0.67); I² = 0%

Test for overall effect: Z = 4.65 (P < 0.00001)

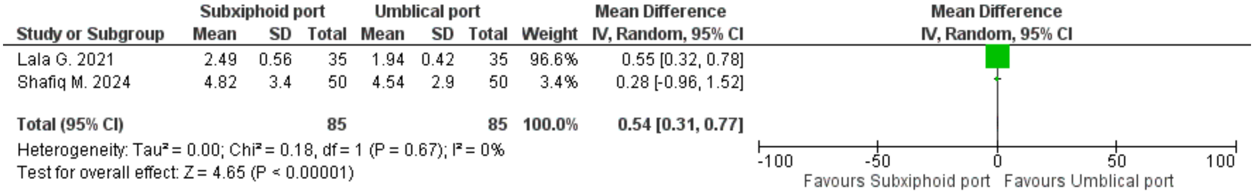

Figure S3: Forest plot of Post-operative pain at 48 hours

Study or Subgroup	Subxiphoid port Mean	SD	Total	Umblical port Mean	SD	Total	Weight	Mean Difference IV, Random, 95% CI
Khan M. A. 2021	4.25	0.13	30	2.51	0.2	30	21.2%	1.74 [1.65, 1.83]
Lala G. 2021	3.34	0.48	35	2.49	0.51	35	21.0%	0.85 [0.62, 1.08]
Rauf H. 2024	3.8	0.83	50	5.42	1.16	50	20.6%	-1.62 [-2.02, -1.22]
Shafiq M. 2024	4.14	3.04	50	5.4	3.3	50	16.2%	-1.26 [-2.50, -0.02]
Siddiqui N.A. 2012	3.9	0.85	60	2.4	0.79	60	20.9%	1.50 [1.21, 1.79]
**Total (95% CI)**			**225**			**225**	**100.0%**	**0.32 [-0.70, 1.35]**

Heterogeneity: $Tau^2 = 1.29$; $Chi^2 = 318.65$, $df = 4$ ($P < 0.00001$); $I^2 = 99\%$

Test for overall effect: $Z = 0.62$ ($P = 0.54$)

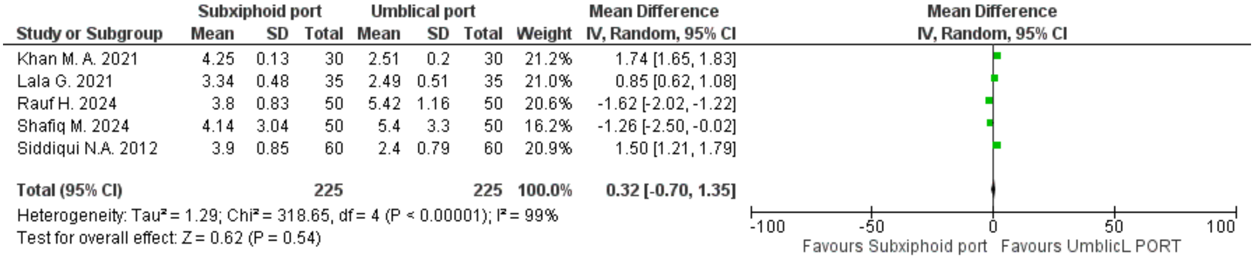

Figure S4: Forest plot of Post-operative pain at 12 hours

Study or Subgroup	Subxiphoid port Mean	SD	Total	Umblical port Mean	SD	Total	Weight	Mean Difference IV, Random, 95% CI
Khan M. A. 2021	5.7	0.09	30	4.38	0.15	30	30.7%	1.32 [1.26, 1.38]
Lala G. 2021	4.09	0.82	35	3.4	0.5	35	22.8%	0.69 [0.37, 1.01]
Rauf H. 2024	5.32	1.08	50	4.08	0.83	50	20.5%	1.24 [0.86, 1.62]
Shafiq M. 2024	4.9	3.2	50	4.38	3.18	50	4.7%	0.52 [-0.73, 1.77]
Siddiqui N.A. 2012	4.6	0.94	60	3.5	1.05	60	21.3%	1.10 [0.74, 1.46]
**Total (95% CI)**			**225**			**225**	**100.0%**	**1.08 [0.78, 1.37]**

Heterogeneity: Tau² = 0.07; Chi² = 17.15, df = 4 (P = 0.002); I² = 77%
Test for overall effect: Z = 7.20 (P < 0.00001)

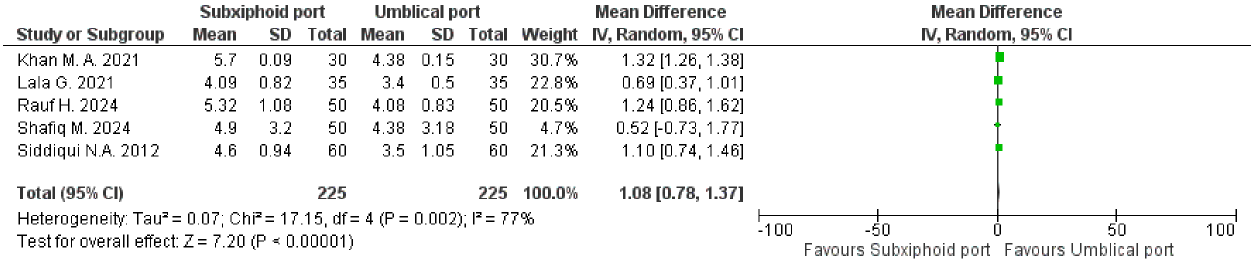

Figure S5: Forest plot of Post-operative pain at 06 hours

Study or Subgroup	Subxiphoid port Mean	Subxiphoid port SD	Subxiphoid port Total	Umblical port Mean	Umblical port SD	Umblical port Total	Weight	Mean Difference IV, Random, 95% CI
Khan M. A. 2021	5.7	0.09	30	4.38	0.15	30	90.2%	1.32 [1.26, 1.38]
Lala G. 2021	4.09	0.82	35	3.4	0.5	35	0.0%	0.69 [0.37, 1.01]
Rauf H. 2024	5.32	1.08	50	4.08	0.83	50	4.4%	1.24 [0.86, 1.62]
Shafiq M. 2024	4.9	3.2	50	4.38	3.18	50	0.4%	0.52 [-0.73, 1.77]
Siddiqui N.A. 2012	4.6	0.94	60	3.5	1.05	60	4.9%	1.10 [0.74, 1.46]
**Total (95% CI)**			**190**			**190**	**100.0%**	**1.30 [1.22, 1.38]**

Heterogeneity: Tau² = 0.00; Chi² = 3.10, df = 3 (P = 0.38); I² = 3%

Test for overall effect: Z = 31.83 (P < 0.00001)

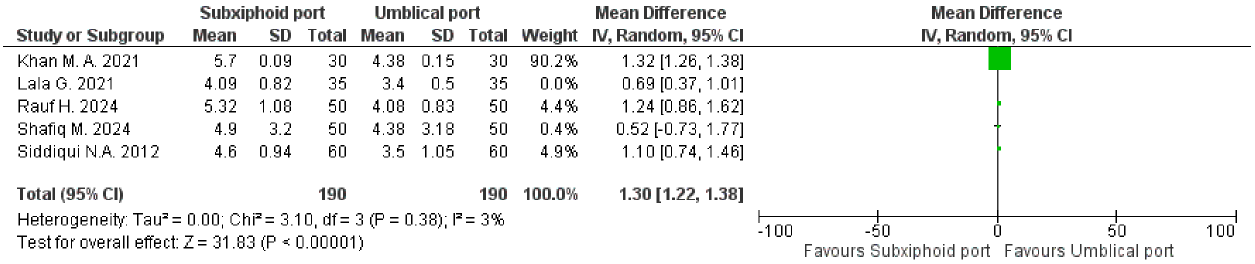

Figure S6: Forest plot of Leave-one out analysis of Post-operative pain at 06 hours

	Subxiphoid port			Umblical port				Mean Difference
Study or Subgroup	Mean	SD	Total	Mean	SD	Total	Weight	IV, Random, 95% CI
Lala G. 2021	5.26	0.66	35	4.14	0.77	35	29.5%	1.12 [0.78, 1.46]
Rauf H. 2024	6.72	1.05	50	5.68	1.15	50	28.0%	1.04 [0.61, 1.47]
Shafiq M. 2024	4.6	3.2	50	5.48	2.8	50	15.2%	-0.88 [-2.06, 0.30]
Siddiqui N.A. 2012	5.9	1.1	60	4.1	1.5	60	27.3%	1.80 [1.33, 2.27]
**Total (95% CI)**			**195**			**195**	**100.0%**	**0.98 [0.35, 1.61]**

Heterogeneity: Tau² = 0.32; Chi² = 18.93, df = 3 (P = 0.0003); I² = 84%
Test for overall effect: Z = 3.04 (P = 0.002)

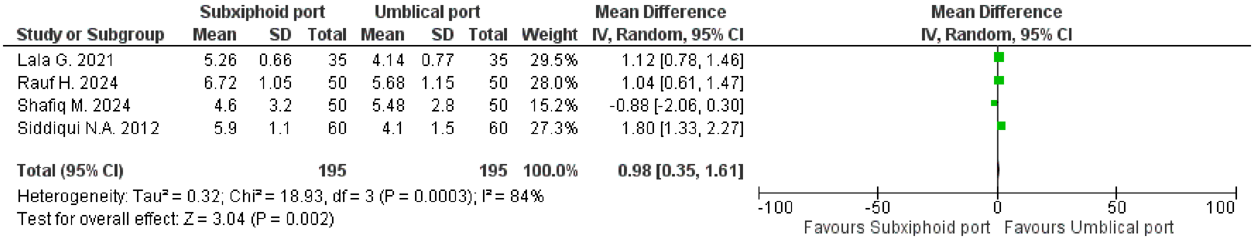

Figure S7: Forest plot of Post-operative pain at 01 hour

Study or Subgroup	Subxiphoid port Mean	SD	Total	Umblical port Mean	SD	Total	Weight	Mean Difference IV, Random, 95% CI
Lala G. 2021	5.26	0.66	35	4.14	0.77	35	37.1%	1.12 [0.78, 1.46]
Rauf H. 2024	6.72	1.05	50	5.68	1.15	50	32.4%	1.04 [0.61, 1.47]
Shafiq M. 2024	4.6	3.2	50	5.48	2.8	50	0.0%	-0.88 [-2.06, 0.30]
Siddiqui N.A. 2012	5.9	1.1	60	4.1	1.5	60	30.5%	1.80 [1.33, 2.27]
**Total (95% CI)**			**145**			**145**	**100.0%**	**1.30 [0.87, 1.74]**

Heterogeneity: $Tau^2 = 0.10$; $Chi^2 = 6.72$, df = 2 (P = 0.03); $I^2 = 70\%$

Test for overall effect: Z = 5.88 (P < 0.00001)

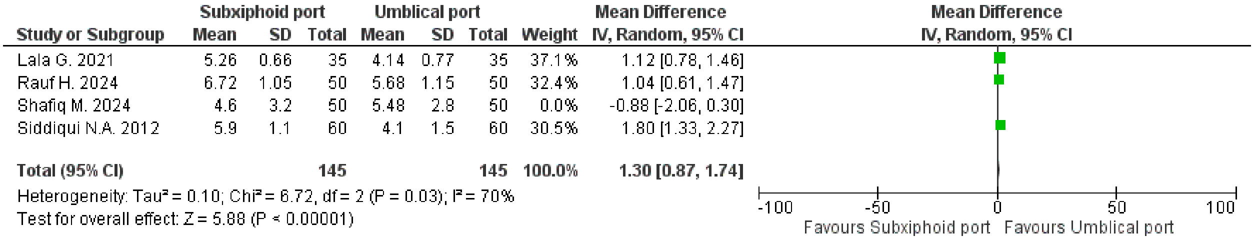

Figure S8: Forest plot of Leave-one out analysis of Post-operative pain at 01 hour

	Subxiphoid port		Umblical port			Risk Ratio
**Study or Subgroup**	**Events**	**Total**	**Events**	**Total**	**Weight**	**M-H, Random, 95% CI**
Rauf H. 2024	7	35	1	35	46.1%	7.00 [0.91, 53.95]
Shaikh B. 2021	5	314	18	314	53.9%	0.28 [0.10, 0.74]
**Total (95% CI)**		**349**		**349**	**100.0%**	**1.23 [0.05, 30.17]**
Total events	12		19			

Heterogeneity: Tau² = 4.70; Chi² = 8.04, df = 1 (P = 0.005); I² = 88%
Test for overall effect: Z = 0.13 (P = 0.90)

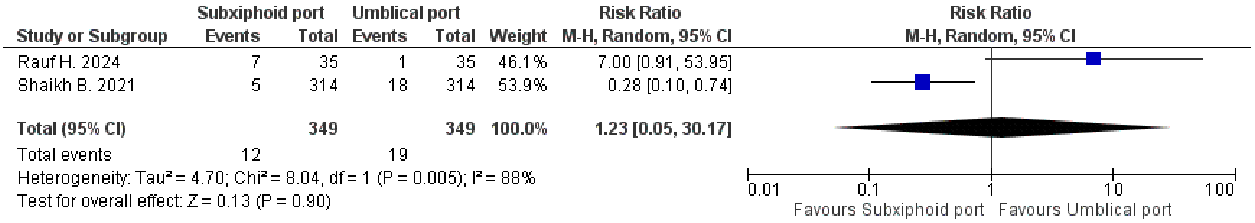

Figure S9: Forest plot of Port Site Infection

Study or Subgroup	Subxiphoid port Mean	SD	Total	Umblical port Mean	SD	Total	Weight	Mean Difference IV, Random, 95% CI
Rauf H. 2024	3.98	0.82	35	3.04	0.81	35	66.6%	0.94 [0.56, 1.32]
Shafiq M. 2024	4.4	2.1	50	4.08	1.97	50	33.4%	0.32 [-0.48, 1.12]
**Total (95% CI)**			**85**			**85**	**100.0%**	**0.73 [0.16, 1.31]**

Heterogeneity: Tau² = 0.09; Chi² = 1.89, df = 1 (P = 0.17); I² = 47%
Test for overall effect: Z = 2.51 (P = 0.01)

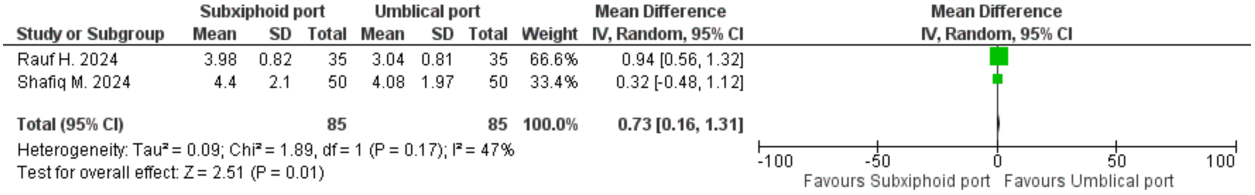

Figure S10: Forest plot of Post-operative Hospital Stay
